# A Variation of Observation-to-imitate Plus Practice Intervention to Decrease Latency to Exercise Initiation and Increase Exercise Completion in Neurological Patients

**DOI:** 10.1298/ptr.25-E10388

**Published:** 2026-07-07

**Authors:** Darci D. DAVIS, Kayla R. RANDALL

**Affiliations:** Psychology Department, Georgia Southern University, USA

**Keywords:** Action observation, Prompt, Neurological disorders, Imitation

## Abstract

**Objectives:**

Some patients referred to physical therapy demonstrate deficits in initiation and completion of physical therapy exercises during appointments. These challenges may create a barrier to benefitting from therapy. We evaluated the effect of observation-to-imitate plus practice (OTI + PP) on decreasing latency to exercise initiation and increasing exercise completion for 2 participants referred for inpatient rehabilitation physical therapy.

**Methods:**

We used an AB multiple-baseline single-case research design to compare the OTI + PP intervention to each participant’s baseline condition for their latency to exercise initiation and completion.

**Results:**

Visual inspection indicates some improvement for 1 of the 2 participants to produce more consistent responding during OTI + PP, but there is an insufficient demonstration of a functional relation due to the small number of participants and lack of effect using visual analysis. The percentage of all nonoverlapping data indicates moderate to large effect sizes of OTI + PP to decrease latency to exercise initiation and increase completion.

**Conclusions:**

It is possible that OTI + PP could decrease latency to exercise initiation and increase completion, but individual variability, insufficient replication, and the small number of participants could limit conclusions and should be considered.

## Introduction

Patients with neurological disorders (e.g., traumatic brain injury, dementia) who have experienced an additional adverse health event (e.g., stroke) may require referral to physical therapy (PT) due to deficits in motor function^1)^. Rehabilitation is a process by which patients relearn necessary skills and behaviors that are currently deficient in their motor repertoire^2)^. One strategy to bring about efficient rehabilitation for patients is to provide a motor template by using live action modeling to demonstrate the desired exercise (i.e., imitation). One intervention, observation-to-imitate plus practice (OTI + PP), has been shown to produce significantly positive outcomes for functional movement in stroke patients and is an enhancement to conventional PT^3)^. OTI + PP is similar to action observation (AO) therapy, but with the exception of using a therapist as the model rather than a video model with additional practice opportunities^3)^.

The neurophysiological mechanism for the efficacy of OTI + PP is AO because it requires a patient’s use of mirror neurons^4)^, and has been suggested to be rooted in mirror neuron theory^5,6)^. AO produces substantial gains in the excitability of the corticospinal pathway when patients engage in imitation and continued practice^3)^. Previous research has shown that various combinations of AO, OTI, with and without practice can produce significant positive effects on increases in functional living skills. AO specifically shows significant effects in terms of increasing functional daily life, including arm and hand motor function, walking ability, gait velocity, and performance in activities of daily living^7,8)^. Research has suggested that AO treatments that use live models are superior to video models because of greater activation in the primary motor cortex^9)^. This finding is supported in research with differing theoretical backgrounds. For instance, a strong parallel to the OTI + PP intervention comes from the behavioral psychology literature, which uses a sequence of systematic model prompts and observational learning to teach imitation to individuals with autism spectrum disorder, another type of neurological disorder^10)^. In one study, video models were compared to live models to promote imitation; participants showed better performance with live models and repeated prompts and practice than in the video model condition^10)^.

Despite the promising nature and convergent evidence across psychological and neuroscientific fields for AO interventions, there has been a large amount of variability in AO protocols, making interpretations about the exact best procedures challenging. Some research has questioned if thorough evaluation is possible because of the varying types of protocols for AO, including differences in (a) content of the videos, (b) duration of video and imitations, and (c) treatment dosages^8)^. For example, some studies have included patients watching videos or live models with varying durations of 50 s to 20 min and practicing the exercises for a range of 2–30 min^7,11)^. Further, there is perhaps less research and consistency in the implementation of an OTI + PP protocol across studies. For example, some studies described showing patients the exercise for a range of 1–2 min but did not describe the extent to which the PTs repeated the model in a systematic fashion. In addition, some OTI + PP protocols have had patients observe the therapist for variable amounts of time, including a duration of 4–30 min.

These subtle differences across studies make interpretations about the most efficacious OTI + PP intervention challenging. In addition, no standardization for treatment parameters has been published due to the varying nature of interventions based on AO therapy. Further, systematically addressing compliance with PT exercises can promote positive outcomes for patients^12,13)^. Toward that aim, we present a single-case research design where we implemented a variation of OTI + PP by the implementation of repeated and systematic AO across each PT session to determine if this could decrease latency to exercise initiation and completion of exercises.

## Methods

### Participants and setting

Prior to recruitment, we obtained approval through the university and hospital institutional review boards (IRB). (University IRB H25207; Hospital IRB 24-030). We recruited patients who were at least 18 years of age, demonstrated a deficit in gross-motor skills, and were referred for PT in an inpatient rehabilitation facility. We recruited 3 participants; however, 1 patient was discharged early. Therefore, we had 2 participants who completed baseline and intervention conditions. Our first participant, Jerry (pseudonym), was a 52-year-old Black male diagnosed with an aneurysmal subarachnoid hemorrhage with no deficits in verbal language and communication. Our second participant, Nancy (pseudonym), was a 70-year-old White female diagnosed with a stroke on the left frontal and bilateral parietal lobes, hypertension, dementia, gastroesophageal reflux disease, and hyperthyroidism. We trained 2 PTs to implement all sessions according to the protocol. The first implementer, Haley (pseudonym), was a 27-year-old Asian female with a doctorate in PT, 2 years and 4 months of experience, and expertise in acute trauma care (cardiac, neurological, and medical intensive care unit), as well as outpatient neurological, vestibular, and orthopedic care. The second implementer, Niles (pseudonym), was a 37-year-old White male with a doctorate in PT, 9 years of experience, and expertise in outpatient worker’s compensation and orthopedics, acute care, and outpatient day rehabilitation program.

All sessions were conducted in an inpatient rehabilitation facility located inside a 384-bed hospital in the southeastern United States. All sessions were conducted within the patients’ regularly scheduled PT appointments, occurring between 7:30 AM and 2:30 PM for 6 days a week. Across all sessions, we used a password-secure laptop equipped with a recording webcam for data collection purposes. Following each video-recorded session, the first author scored all sessions for the primary dependent variables.

### Experimental design and response measurement

We used an AB multiple-baseline across participants single-case research design^14)^. The experimental logic of this design is composed of a time-lagged and staggered introduction of the independent variable and compares within- and across-participant responses of baseline and intervention conditions. All sessions, regardless of baseline or intervention, were considered at a minimum standard of care; therefore, no patients who had extended baselines experienced a diminished treatment during baseline. We sequentially assigned patients to a baseline tier upon their enrollment in treatment and consent to participate in the study.

For response measurement, we had 2 primary dependent variables: latency to initiation and completion of exercise. We defined latency to initiation of each exercise as any instance following the PT’s model in which the participant began to move in a manner that was consistent with an attempt to imitate the PT’s model. We measured latency to initiation by determining the duration in seconds by collecting the inter-response time between the end of the PT’s model and the time at which the participant initiated the exercise. We selected latency to initiation as the measure to capture discrete potential changes in participant willingness to comply with exercise, even if they were unsuccessful at exercise completion (i.e., a measure of motivation rather than skill deficit). We defined completion of an exercise when a patient had completed imitation and point-to-point correspondence within 7.62 cm of the PT’s model. If we observed an exact match, we considered this 100% completion. We measured partial imitation as when the participant’s behavior approximated the model, but the placement of their body was not within 7.62–15.24 cm of the model. If we observed a partial match with some variability in accuracy, we considered this 50% completion. Finally, we measured no imitation, which we defined as the participant following their movements not resulting in any point-to-point correspondence or there being more than 15.24 cm between the model and the imitator. If we observed either no attempt or an imitation that did not have any match within 15.24 cm of the model, we considered this 0% completion. We measured completion in this manner due to potential variability in exercise completion and inadvertent errors made regarding subtle and nuanced movements during direct observation. We attempted to balance allowing individual variability in movements while maintaining pre-specified cutoff points objectively applied across participants. In collaboration with the PTs who implemented the sessions, we developed these definitions. We defined a model prompt as a model (i.e., demonstration of a specific exercise) accompanied by a verbal prompt (i.e., a description of the specific exercise).

### Baseline

During baseline, the PT followed their standard-of-care procedures and implemented verbal requests for patients to complete each exercise (e.g., march, leg kicks, stand up). Because we encouraged the PTs to follow “baseline-as-usual” procedures, there was slight variation in the number of exercises, other types of prompts (e.g., tactile cues, verbal prompts), and positive or corrective feedback across each session.

### Intervention

For the intervention, we implemented a variation of OTI + PP. Specifically, we asked the PT to ensure the patient was looking at the therapist. The PT then named the exercise while modeling the behavior. For example, the PT stated, “March,” and modeled marching by lifting and lowering legs in place. The PT waited for initiation or completion of the behavior. If the participant did not initiate the behavior within 25 s, the PT modeled the behavior a second time. If the participant again did not initiate the behavior within 25 s, the PT modeled the behavior for a third time. The PT then waited for the initiation or completion of the behavior. If the participant initiated or completed the behavior within 25 s of either the first, second, or third prompt, the PT provided a positive feedback in the form of behavior-specific praise. If the participant did not complete the exercise within 25 s of the third prompt, the PT selected a new exercise to model and did not provide positive praise. Similar to the development of the operational definitions for completion of exercise, we selected 25 s as the interval based on input from the PTs implementing the intervention and previous anecdotal clinical observations.

## Results

### Visual analysis

We applied visual inspection, which is the primary and hallmark evaluation of single-case research. Figure 1 displays closed data points indicating latency to initiation (panels 1 and 3) and completion of exercises (panels 2 and 4) across participants and trials in baseline and OTI + PP, respectively. Gray data points on the panels displaying latency indicate a trial in which the participant did not respond (as opposed to completing exercise with low latency). We observed moderate to short and variable latencies to initiation across participants in baseline. Upon introduction of OTI + PP, we observed a decrease in overall variability with more consistent low to zero latencies for Nancy. For Jerry, we observed more frequent zero latencies in initiation but no improvement in completion, suggesting that zero latencies occurred because of no attempt to complete the exercise. No significant differences in the reduction of latency can be observed between baseline and intervention across either participant. During baseline, we observed moderate to high but highly variable percentage completion of exercises across both participants. For Nancy, we observed more consistently high percentages of exercise completion upon the introduction of OTI + PP, whereas for Jerry, we observed a decrease to consistently zero percentages of completion of exercises, indicating a potential countertherapeutic effect.

**Fig. 1.**
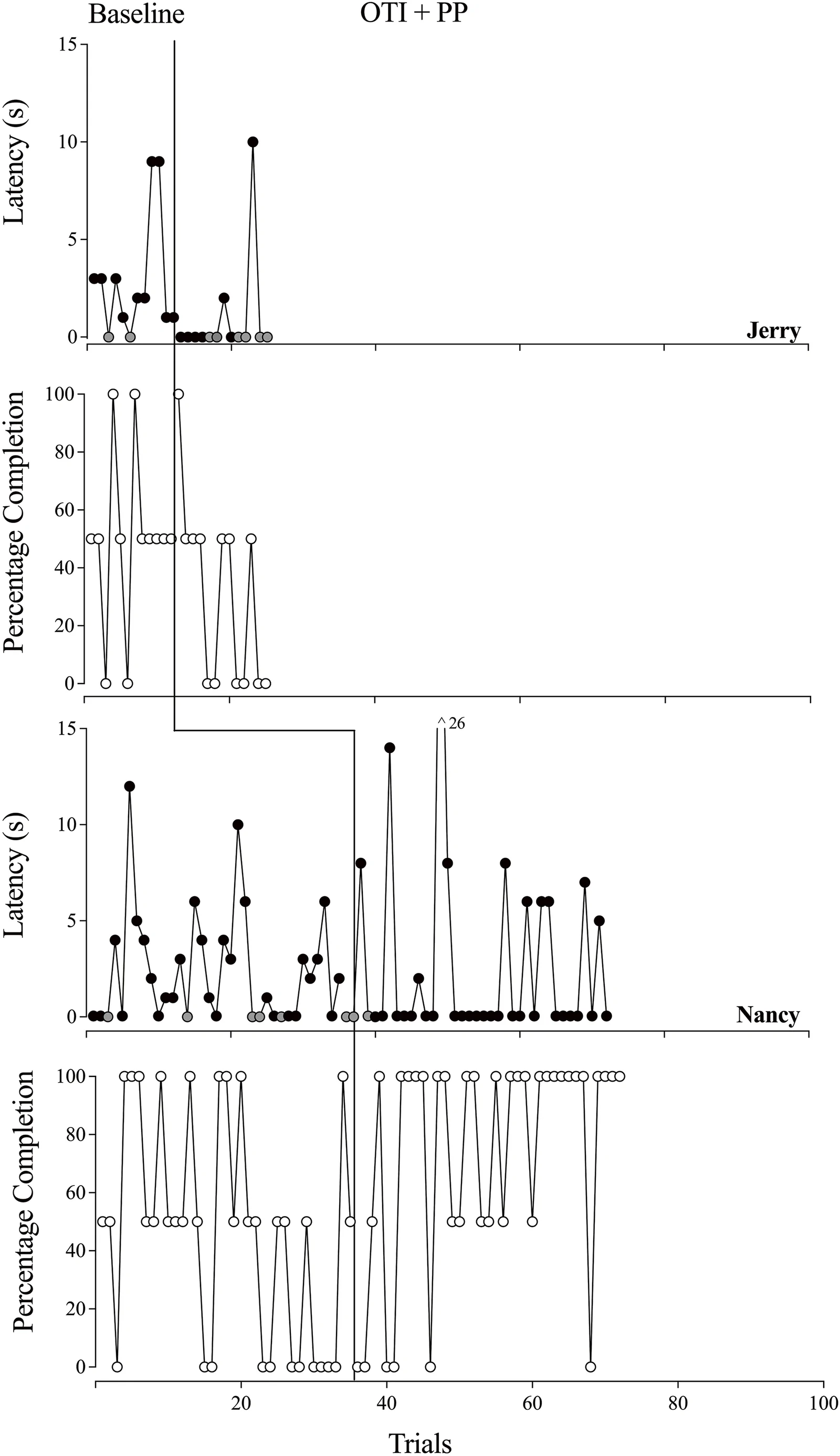
Latency to exercise initiation and percentage of exercise completion across participants. (Jerry and Nancy are pseudonyms). OTI+PP, observation-to-imitate plus practice

### Statistical analyses

We used the single-case effect size calculator^15)^ to calculate the percentage of all nonoverlapping data (PAND)^16)^, which is a way to calculate an effect size of change by considering all of a participant’s data between baseline and intervention conditions. For Nancy, the decrease in latency to exercise initiation PAND was 0.88, and completion of exercise PAND was 0.52, indicating large to moderate effects of OTI + PP to decrease latencies to exercise initiation and increase exercise completion, respectively. For Jerry, the decrease in latency to exercise initiation PAND was 0.69, and completion of exercise PAND was 0.69, indicating moderate effects of OTI + PP to decrease latencies and increase exercise initiation.

## Discussion

The results indicate that OTI + PP may be considered a beneficial addition to conventional PT to potentially improve inconsistent latencies to initiation and exercise completion. However, caution is warranted given the small number of participants and overall variability in responding with an absence of a strong functional relation across both dependent variables. In addition, there is some evidence to suggest that although there were decreases in latency, there was an overall decrease in exercise completion for Jerry. This suggests that for this participant, regardless of shorter latencies to exercise initiation, it did not produce higher or consistent levels of exercise completion, making a questionable, and even potentially contraindicated, effect for Jerry. There are discrepant outcomes between the 2 dependent variables, which highlight how different outcome measures may impact conclusions about an intervention. Although different measures may be important to ascertain an entire picture of progress, caution is warranted, and a limitation is imposed in our study because the 2 outcome measures indicate different conclusions. Specifically for the completion measure, it is possible that the research methodology used to code completion inadvertently diminished or limited the observation of client progress. That is, there may have been consistent improvements in partially completed movements, but this may not have been reflected in the data.

Even so, these data contribute additional evidence that AO, through live modeling, may be helpful to decrease long durations of noncompliance to initiate and complete PT exercises during therapy. We included a secondary statistical analysis to supplement visual inspection to allow for potentially greater visibility of single-case studies in future meta-analyses, which rely on effect sizes^17)^. However, it is important to note that although our secondary statistical tests indicated moderate to large effects, visual analysis of the data does not demonstrate a strong or reliable functional relation. Reliance on visual inspection should be the primary means of determining functional relations; therefore, our conclusion is that OTI + PP may indicate some potential but small effect for 1 out of 2 patients.

Another consideration for the results is the manner in which we defined and measured completion of exercises. Our primary reliance on operational definitions, direct observation, and measurement came from our philosophical underpinnings and background in the behavior analytic literature, which relies on observation of discrete instances of behavior^18)^. It is typical to define behaviors and then determine whether they did or did not occur within the boundary conditions of some criterion of occurrence. However, this level of precision can be difficult to capture by observers^19)^. In an attempt to balance the precision of accuracy of imitation for patients while allowing variability in responding, we determined, in collaboration with the PTs, the amount of allowable variability while still qualifying whether an exercise was completed. As such, we used these same types of measures, in collaboration with the PTs, to allow for total, partial, or no completion. Although these definitions of completion were developed following observation of patients, the cutoff points for partial or no completion were arbitrarily selected and have not been clinically verified. Further, there may be compromised clinical significance of how we measured exercise completion because it relied on very small differences in movements (e.g., 7.62–15.24 cm); this may not result in meaningful functional improvement for patients. However, we used this intervention in the context of standard-of-care therapy, which was focused on functional improvement. In addition, by using small movements, we could more precisely identify the impact of intervention on motivation, which helped control for lack of completion due to response effort or difficulty. Even so, the overall response definitions of exercise completion may need modification to observe functional improvement in future patients. Similarly, the 25-s interval prior to prompting was selected based on clinical observation in collaboration with the PTs implementing intervention; however, this interval was arbitrarily selected and could have inadvertently placed a ceiling on patient completion of exercise. With these limitations, caution regarding the conclusions of the results is warranted given the potential drawbacks of a lack of clinically meaningful changes in the patient’s behavior.

The lack of replication across participants may be due to a lack of functional similarity in deficits and needs across participants. Although both participants were referred to the same inpatient rehabilitation therapeutic unit and had similar exercise goals (e.g., marches, sit to stand), there were significant differences in their individual characteristics, diagnoses, and language delays. In a multiple-baseline-across-participants design, it is important to include participants that have similar functional deficits to demonstrate replication across participants. Although we attempted to recruit participants that had similar needs, there were potentially large differences in their baseline functioning and characteristics that led to disparate responses to intervention. Future research may consider a pre-baseline assessment for attending to a therapist and levels of latency to completion and compliance prior to enrolling patients.

It is possible that incorporating OTI + PP may allow for more consistent responding and better demonstration of improvement for exercise. This approach, with underpinnings in mirror neuron therapy in neuroscience and systematic prompting in behavioral psychology, may produce small but positive outcomes for patients during PT appointments and can have moderate to large effects for decreases in latency to initiation and completion of exercises; however, this conclusion should be considered in light of visual analysis that may indicate little to no effect of the intervention.

## Conclusions

Overall, we found that OTI + PP could produce more consistent decreases in latencies to initiation and exercise completion in some patients; however, visual inspection of participants’ responding in this study does not produce a strong or apparent functional relation between OTI + PP and meaningful, impactful decreases in latencies to exercise initiation and exercise completion. Notwithstanding, there have been many applications of interventions that are conceptually and topographically similar to OTI + PP that have strong empirical support for improving initiations of imitation and completion of skills. However, this study is limited due to the number of participants and potential differences between participants to produce replication across patients. Additional limitations of this study include questionable confidence in the independent variables because we programmed baseline-as-usual conditions, which involved several features of the intervention (e.g., tactile cues, prompting, and feedback); thus, it is difficult to clarify the exact impact of the intervention since many of these elements were freely included in the baseline condition. However, one aspect of the baseline that we did not include was the systematic implementation of these variables; thus, differences between baseline and intervention conditions could be because of the nature of the implementation of the intervention. In addition to the potential uncontrolled nature of baseline conditions, there were also several components embedded as part of the intervention package. It is relatively common to have several components in a behavioral intervention; however, it is not possible to know which component produced any change. Without a component analysis, it is not possible to identify the controlling variables. Future research should consider specific treatment components for evaluation of OTI + PP as an intervention using AO as a treatment for increasing rehabilitation.

## Data Availability

The data supporting the findings of this study are available from the corresponding author upon reasonable request.
